# Carboxymethyl cellulose assisted reforming of poly acrylic acid co methyl methacrylate composite for wastewater treatment and effective hosting of antimicrobial silver

**DOI:** 10.1038/s41598-025-86214-5

**Published:** 2025-02-08

**Authors:** Ahmed Hamdy, Hassan Nageh, S. A. Hassan, Mohamed A. Mekewi, Atef S. Darwish

**Affiliations:** 1 Department of Chemistry, Faculty of Science, Ain Shams University, Cairo, 11566 Egypt; 2 Nanotechnology Research Centre (NTRC), The British University in Egypt, Cairo, 11837 Egypt; 3 Faculty of Dentistry, The British University in Egypt, Cairo, 11837 Egypt

**Keywords:** Carboxymethyl cellulose, Poly (acrylic acid-co-methyl methacrylate), Adsorption of safranin, Ag particles, Antimicrobial, Microbiology, Environmental sciences, Chemistry, Materials science

## Abstract

Herein, novel polymer composite is fabricated by hybridizing poly (acrylic acid-co-methyl methacrylate) filaments with carboxymethyl cellulose, which efficiently reorients and strictly ties the fibrous chains to form polymeric units of plate-like morphology. This innovative hybrid polymer composite is analyzed using XRD, FT-IR, swelling and contact angle studies, DLS, AFM, and SEM. Removal efficiency of such polymer composite is scrutinized in colored wastewater treatment. Langmuir and pseudo-first-order kinetic models best describe safranine dye removal from wastewater, adopting exothermic adsorption progression with elevated capacity (~ 59.47 mg/g) and accelerated rate (~ 1.06 h^− 1^). Such polymer composite exhibits persistent removal efficiency of ~ 90% within 10 min for five consecutive cycles. Hybrid polymer composite is good candidate platform for hosting Ag particles to heighten their antimicrobial activity against *Escherichia coli* and *Staphylococcus aureus*, far exceeding 75% reduction. Future studies on applicability of oxygen-rich polymer composites in wastewater treatment and disinfection are optimistic and extremely competent.

**Supplementary Information:**

The online version contains supplementary material available at 10.1038/s41598-025-86214-5.

## Introduction

Discharging industrial effluents into the environment poses a serious threat to aquatic life and the food chain by introducing heavy metals, organic pollutants, and dyes, which can lead to severe infections in human beings^1,2^. Safranin dye, a water-soluble cationic dye, is particularly significant and extensively used in both the textile industry and microbiology^3^. As is reported in the literature, prolonged exposure to safranin dye can result in eye irritation, corneal and conjunctival degeneration, skin irritation, throat discomfort, and various long-term health issues affecting the respiratory and digestive systems^2,4^. Various methods are employed to treat dye-contaminated wastewater, including adsorption techniques^5,6^, electrocoagulation^7^, membrane filtration^8^, enzymatic degradation^9^, and advanced oxidation processes^7^. In addition, flocculation process issues as one of the remarkable technologies in wastewater treatment, whereas hybrid polymer composites of reasonable hydrophilic nature, like polyacrylamide-grafted polyvinyl pyrrolidone and polyacrylamide-grafted-gum ghatii, are supposed as good candidate flocculants for the removal of hazardous materials from contaminated water^10,11^. Indeed, the adsorption approach remains a strong contender for wastewater depollution when compared to other purification techniques^12^. Its enduring popularity is owed to its simplicity, convenience, high efficiency, low cost, and wide applicability. These attributes collectively make it a reliable and effective method for removing pollutants from wastewater^2,13^.

Poly acrylic acid (PAA) is a low-cost hydrophilic biocompatible polymeric hydrogel of poor toxicity. PAA possesses advanced optical properties and high resistance to weathering^14^. According to the literature, PAA is a promising agent for obliteration of organic pollutants from industrial wastewater^15^. Of particular interest, PAA can be effectively hybridized with various polymeric segments to enhance its mechanical properties and adsorption capacity. Consequently, PAA finds significant use in a variety of environmental and biological applications^16,17^.

On the other hand, polymethyl methacrylate (PMMA), as a hydrophobic polymer, exhibits low toxicity, strong absorption capabilities for organic pollutants, and excellent mechanical properties. Therefore, it finds applications in various industrial processes such as wastewater treatment, food packaging, and pharmaceuticals^14,18^. Options proposed in the literature suggest that the electronic, mechanical, and swelling properties of poly acrylic acid can be optimized by hybridizing it with hydrophobic polymers like PMMA, wherein hydrophilic and hydrophobic monomers are copolymerized in different ratios^19–21^. To the best of our knowledge, relatively few studies in the literature focus on the use of poly (AA-co-MMA) as an effective depolluting agent in wastewater treatment.

Recently, carboxymethyl cellulose (CMC) has been the most efficient additive material to various hydrogel adsorbents to improve their efficiency toward the removal of organic pollutants from wastewater compared to other cellulose derivatives^22–24^. This consensus refers to acquisition of CMC to superb adsorption affinity, biodegradability, and biocompatibility of various hydrogels, particularly polyacrylic acid^22^. In the past few years, the adsorption affinity of PAA hydrogel has been significantly enhanced when being conjugated with various inexpensive biopolymers such as carboxymethyl cellulose, chitosan, alginate, gelatin, guar gum, and xanthan gum^22^. Such modification endows PAA hydrogel with efficacious performance in removing a wide range of dyes from industrial wastewater^2,24^. Inspection of literature, copolymerization of vinyl monomers, in particular, acrylic acid, acrylamide, acrylonitrile, and hydroxyethyl methacrylate, hand in hand with their grafting onto natural polymers, especially cellulose and its derivatives, has become a pivotal approach for manufacture of hydrogels and superabsorbent polymers^25,26^. For instance, sodium carboxymethyl cellulose-grafted-polyacrylic acid exhibits a preeminent swelling capacity of ~ 545 g/g in distilled water and about 50 g/g in an electrolytic solution containing NaCl (0.9%w/v)^25^. In addition, CMC-grafted-poly (sodium acrylate)/kaolin possesses advanced swollen gel strength with super absorbing power and poor salt-sensitivity^26,27^. To our knowledge, fabrication of CMC/p(AA-co-MMA) hybrid polymer composite and investigation of its adsorption performance as well as removal efficiency toward organic pollutants from wastewater have not been reported in the literature yet.

Embedding silver particles into a polymeric matrix promises immense potential in the field of antimicrobial therapy as metallic silver acquires superior destructive power against the membrane and cytoplasm of Gram-negative and Gram-positive bacteria^11,28^. Besides, incorporation of Ag particles into polymeric moiety devotes controllable releasement of Ag species, thus prohibiting cytotoxicity effects on human beings^29^. In this respect, the present study goes through hosting Ag^+^ ions into specialized polymeric matrices of hydrophilic/hydrophobic character thereby facilitating the photolytic reduction process to form metallic Ag particles.

The current study aims to fabricate a novel hybrid polymer composite based on the hybridization of poly (acrylic acid-co-methyl methacrylate) matrix with carboxymethyl cellulose and recognize its depolluting performance against dyeing wastewater. Another objective of this study is to exploit such hybrid polymer composite for hosting Ag particles and distinguishing their disinfection performance against pathogenic microorganisms like *Staphylococci aureus* (*S. aureus*) and *Escherichia coli* (*E. coli*).

## Materials and methods

### Materials

Carboxymethyl cellulose (CMC), acrylic acid (AA, 99%), methyl methacrylate (MMA, 98%), potassium persulfate (KPS, 99%) initiator, N, N-methylene-bis(acrylamide) (NMBA, 99%) cross-linker, dimethyl sulfoxide and silver nitrate were purchased from Sigma-Aldrich (St. Louis, MO, USA). Safranin red dye (C_20_H_19_N_4_Cl, MW 350.85 g/mol) was procured from TATA Chemicals Ltd. (Mumbai, India) and used to prepare 1000 mg/L (pH: 7.8) stock solution, being accredited via a standard calibration curve taken at wavelength 520 nm. Deionized water was used throughout this study.

### Preparation of p(AA-co-MMA) copolymer

To prepare the p(AA-co-MMA) copolymer, given amounts of AA (1.57 ml) and MMA (1.40 ml) were dissolved in 15 ml mixed solvent (deionized water: dimethyl sulfoxide of volume ratio 2:1), where the molar feed ratio of AA with respect to MMA monomer achieved 50/50. The reaction mixture was stirred at room temperature and then appropriate amounts of KPS initiator as well as NMBA cross-linker were added sequentially registering 1% w/w and 5% w/w, respectively, concerning the total monomers masses. The concentrations of KPS and NMBA in the reaction mixture were equivalent to 8.8 × 10^− 3^ M and 6.5 × 10^− 3^ M, respectively. The mixture was ultrasonically stirred at 60 °C for 8 h to ensure complete gelation. The obtained copolymer was then separated, washed several times with deionized water and finally freeze-dried under vacuum for 24 h.

### Preparation of CMC/p(AA-co-MMA) hybrid polymer composite

The aforementioned procedure was followed in the preparation of p(AA-co-MMA) hybridized with CMC. Similar amounts of AA and MMA monomers were mixed and dissolved in 15 ml solution containing deionized water and dimethyl sulfoxide with a volume ratio of 2:1. The molar feed ratio of AA to MMA monomer is designed to reach 50%. Afterwards, an appropriate amount of CMC (0.15 g) was added to the obtained reaction mixture, recording 5% w/w concerning the total mass of monomers. The reaction mixture was stirred in an ultrasonic bath at room temperature for 1 h and then appropriate amounts of KPS as well as NMBA were added sequentially. The concentrations of KPS and NMBA were analogues to those mentioned above in “Preparation of p(AA-co-MMA) copolymer” section. The given reaction mixture was vigorously stirred at 60 °C for 8 h. The obtained hybrid polymer composite was then filtered, washed with deionized water, and freeze-dried for 24 h under vacuum.

### Preparation of metallic silver particles embedded onto p(AA-co-MMA) copolymer and CMC/p(AA-co MMA) hybrid polymer composite using photo-reduction method

A proper amount of silver nitrate (0.14 g) was dissolved in a 15 ml mixed solvent of deionized water and dimethylsulfoxide (2:1 volume ratio) containing NMBA (6.5 × 10^−3^ M). Known amounts of AA (1.57 ml) and MMA (1.40 ml) were then added to the solvent mixture and stirred in ultrasonic bath for 2 h at room temperature. The mass ratios of the added monomers and cross-linker resembled those found during the synthesis of p(AA-co-MMA). The obtained mixture was photolyzed by UV-light irradiation of wavelength 365 nm for 1 h to affirm complete reduction of Ag^+^ ions, as verified by Katime et al.^29^. The formed brownish suspension was then treated with KPS, where the concentration of the initiator became 8.8 × 10^−3^ M offering a mass ratio equivalent to 1% w/w with respect to the total monomers masses. The obtained reaction mixture was ultrasonically stirred at 60°C for 8 h. Finally, the as-prepared hybrid polymer-based silver composite was collected, washed, and freeze-dried as described above in the synthesis of p(AA-co-MMA). The so-synthesized composite was nominated by “Ag@p(AA-co-MMA)”. The same procedure was followed to prepare the Ag@CMC/p(AA-co-MMA) composite, taking into consideration containing the solvent mixture to CMC (0.15 g), and AA and MMA monomers.

### Characterization

Microstructural analysis of p(AA-co-MMA) and CMC/p(AA-co-MMA) samples was estimated using (i) infrared spectroscopy technique (FT-IR) model (Bruker Vertex 70, Germany) with spectrum in the range 400–4000 cm^−1^, (ii) X-ray diffraction model (Malvern Analytical, Empyrean 3, UK) equipped by a copper anode at 40 keV and 30 mA with intensity scan at ambient conditions in the range 2θ = 5°–80°, (iii) dynamic light scattering (DLS, Malvern zeta sizer-Nano ZS90, United Kingdom) for demonstration of zeta potential distribution curve and average zeta potential value (ζ_av_), as being described elsewhere^1^, and (iv) swelling study, where the as-prepared samples were soaked in deionized water at 25 °C for different time intervals ranging from 0 to 73 h and the amount of absorbed water was recorded using an electronic balance. The percent swelling (S%) was determined using Eq. (1):

1 $${\text{S}}\% = \left[ {\left( {{\text{W}}_{{\rm{t}}} - {\text{W}}_{{\rm{d}}} } \right)/{\text{W}}_{{\rm{d}}} } \right] \times 100$$

Where W_t_ and W_d_ were the weight of the so-synthesized samples at time t and initial dried weight, respectively. Also, the wettability or hydrophilicity of the samples under study was evaluated by measuring the contact angle between polymer surface and water drop with the help of an optical Tensiometer apparatus (KRUSS Model DSA) supplemented with a digital camera.

The morphological analysis of the understudied samples was estimated with the aid of (i) scanning electron microscopy SEM model (Quattro S, Thermo-Scientific, USA), and (ii) atomic force microscope (AFM, Flex axiom Nano surf C3000), where the measurements were detected at room temperature using a special silicon probe of rectangular shape conducting with a resonant frequency of 9 kHz.

### Phenomenological studies of the adsorption of safranine dye over p(AA-co-MMA) copolymer and CMC/p(AA-co-MMA) hybrid polymer composite

The adsorption of safranin dye onto p(AA-co-MMA) and CMC/p(AA-co-MMA) samples was carried out at a specific operating pH value (~ 7.8), concurring with the normal environmental circumstances. The stock solution of safranin dye was diluted to appropriate amounts ranging from 10 to 160 mg/L. In 100 ml conical flask, a proper amount of the polymer sample under study (0.1 g) was added into 50 ml of dye solution of a required concentration. The suspension was left under vigorous magnetic stirring at operating temperature. At given time intervals, 3 ml aliquots were sampled from the examined dye solution and centrifuged at 8000 rpm for 5 min to remove the polymeric material. Afterwards, the supernatant solutions were analyzed using a UV–Vis spectrophotometer (Model Cary 5000 UV–Vis–NIR spectrophotometer with scanning between 200 and 800 nm, Thermo-Fisher Scientific, USA) to detect the remained concentration of safranin dye in solution. The adsorption capacity of safranin dye was calculated as given below,

2 $${\text{q}}_{{\rm{t}}} = \left( {{\text{C}}_{{\rm{o}}} - {\text{C}}_{{\rm{t}}} } \right) \times {\text{V}}/{\text{m}}$$

where q_t_ was the sorption capacity of safranin per unit weight of as-prepared samples at time t (mg/g); C_o_ and C_t_ were the initial and liquid phase concentrations of safranin solution at interval time t (mg/L), being derived from the absorbance values recorded by UV–vis spectrophotometer by the aid of safranin calibration curve, respectively; V was the volume of safranin solution used (L); and m was the mass of the examined p(AA-co-MMA) copolymer and CMC/p(AA-co-MMA) hybrid polymer composite (g). Removal percentage of safranin dye (% R) was given by Eq. (3)

3 $${\text{R}}\left( \% \right) = \left[ {\left( {{\text{C}}_{{\rm{o}}} - {\text{C}}_{{\rm{e}}} } \right)/{\text{C}}_{{\rm{o}}} } \right] \times 100$$

where C_o_ and C_e_ were the initial and final concentrations of safranin dye, respectively in mg/L. Adsorption experiments were performed in triplicate to assure reproducibility and average values were reported. All the adsorption parameters extracted from the various employed adsorption models in the present work were computed using Origin8© software. The statistical analysis was performed using the SPSS software (Statistical Analysis for Social Science, version 19) and the data were tabulated as means ± SD.

#### Isotherm study

Batch adsorption experiments for removal of safranin dye by p(AA-co-MMA) and CMC/ p(AA-co-MMA) were conducted for 3 h at different concentrations of safranin dye in the range from 10 mg/L to 160 mg/L. The temperature during the adsorption process was adjusted to 25°C. At the end of the adsorption process, the produced suspensions were centrifuged and analyzed, as mentioned above, to determine the equilibrium sorption capacities (q_e_, mg/g) and evaluate adsorption isotherms. Different adsorption models were applied to interpret the feasible adsorption mechanism. Freundlich isotherm devoted validity of multilayer sorption on heterogeneous surfaces of indefinite number of binding sites^30^. The linear form of Freundlich equation may be written as:

4 $${\text{log }}{{\text{q}}_{\text{e}}}={\text{log }}{{\text{K}}_{\text{f}}}+{\text{ n log }}{{\text{C}}_{\text{e}}}$$

where C_e_ and q_e_ were the equilibrium concentrations of safranin in solution (mg/L) and the sorption capacity of dye per unit weight of the understudied samples (mg/g), respectively. Freundlich coefficient (K_f_, mg^1 − 1/n^ L^1/n^ g^− 1^) and n were assigned to the adsorption capacity and the adsorption intensity of the understudied polymeric materials, respectively. Langmuir isotherm was a well-interpreted model for monolayer sorption onto nearly homogeneous surfaces, which owed a finite number of binding sites, assuming uniform sorption energies onto the surface with forbidden sorbent transmigration along the surface plane^31^. The linear form of Langmuir was prescribed by the following equation:

5 $${{\text{C}}_{\text{e}}}/{{\text{q}}_{\text{e}}}={\text{1}}/{\text{b}}{{\text{q}}_{\text{m}}}+\left( {\left( {{\text{1}}/{{\text{q}}_{\text{m}}}} \right){\text{ x }}{{\text{C}}_{\text{e}}}} \right)$$

where q_m_ was the monolayer adsorption capacity (mg/g) and b belonged to the adsorption energy (L/g). Also, the perception of Langmuir isotherm was investigated by the dimensionless constant separation term (R_L_) for detecting the nature of adsorption process and is described by Eq. (6)

6 $${\text{R}}_{{\text{L}}} = 1/(1 + {\text{b}}\;{\text{C}}_{{\text{o}}} )$$

Dubinin–Radushkevich equilibrium (D-R) isotherm model was also used for adsorption data fitting to estimate the apparent free energy (E, kJ mol^− 1^) and the theoretical adsorption saturation capacity (q_D_, mg/g)^32^. The D–R isotherm can be linearized as represented in the following equation,

7 $${\text{Ln }}{{\text{q}}_{\text{e}}}={\text{Ln}}{{\text{q}}_{\text{D}}}-\left\{ {{{\left( {{\text{RT Ln }}\left( {{\text{1}}+\left[ {{\text{1}}/{{\text{C}}_{\text{e}}}} \right]} \right)} \right)}^{\text{2}}}/\left( {{\text{2}}{{\text{E}}^{\text{2}}}} \right)} \right\}$$

where R and T were the general gas constant and the adsorption temperature, respectively.

#### Kinetic study

A known amount of the as-prepared polymeric samples (0.1 g) was added into 50 ml dye solution of a concentration of 120 mg/L at different time intervals within the range of 0.25–3 h, and the temperature was sustained at 25°C. The adsorption kinetics were evaluated using both pseudo-first-order^32^ and pseudo-second-order models^33^ for the rate expressions given, respectively, by

8 $$\;\;\left( {\text{a}} \right)\quad {\text{ln }}\left( {{{\text{q}}_{\text{e}}}-{{\text{q}}_{\text{t}}}} \right)={\text{ln }}{{\text{q}}_{\text{e}}}-{{\text{k}}_{1}}{\text{t}}$$

9 $$\left( {\text{b}} \right)\quad {\text{t}}/{{\text{q}}_{\text{t}}}={\text{1}}/\left( {{{\text{k}}_{\text{2}}}{{\text{q}}_{\text{e}}}^{{\text{2}}}} \right)+{\text{t}}/{{\text{q}}_{\text{e}}}$$

where k_1_ and k_2_ represented the pseudo-first-order rate constant (h^− 1^) and pseudo-second-order rate constant (g mg^− 1^ h ^− 1^), respectively.

For p(AA-co-MMA) copolymer and CMC/p(AA-co-MMA) hybrid polymer composite, the likelihood of diffusion of adsorbate species either from the liquid bulk phase up to the adsorbent surface or within the polymeric pore channels may be potentially underpinned. In accordance, intraparticle diffusion approach should be considered and judiciously described by Weber and Morris^34,35^. The intra-particle diffusion rate constants (k_i_, mg g^− 1^ h^− 0.5^) were assessed using Eq. (10) as given below:

10 $${{\text{q}}_{\text{t}}}={{\text{k}}_{\text{i}}}{{\text{t}}^{0.{\text{5}}}}$$

where k_i_ signified the slope of straight-line portions of plot q_t_ vs. t^0.5^. These plots generally had dual nature, i.e., initial, and final linear portions. The initial portions represented the boundary layer diffusion effects while the final portions reflected the result of intra-particle diffusion effects^35^.

#### Thermodynamic study

The adsorption study for the removal of safranine dye wastewater was performed at various temperatures (25°C, 35°C and 50°C) for 3 h at a dye concentration of 120 mg/L. Thermodynamic parameters, ΔH° and ΔS°, were calculated from the slope and intercept of a plot of ln(q_e_/C_e_) against 1/T using the following equation, as discussed in previous studies^36–38^.

11 $${\text{ln }}\left( {{{\text{q}}_{\text{e}}}/{{\text{C}}_{\text{e}}}} \right)={\text{D}}{{\text{S}}^{\text{o}}}/{\text{R}}-{\text{D}}{{\text{H}}^{\text{o}}}/{\text{RT}}$$

where (q_e_/Ce) was nominated by thermodynamic equilibrium constant, T was the temperature (K), R was the general gas constant (kJ mol^− 1^ K^− 1^), ΔH° belonged to the molar enthalpy change (kJ mol^− 1^), and ΔS° is the molar entropy change (kJ mol^− 1^ K^− 1^). Also, ΔG^o^, Gibbs free energy change (kJ mol^− 1^), was investigated using Eq. (12)^38^ as mentioned below,

12 $$\Delta {{\text{G}}^{\text{o}}}=\Delta {{\text{H}}^{\text{o}}}-{\text{ T}}\Delta {{\text{S}}^{\text{o}}}$$

#### Regeneration and reusability studies of the spent polymers under investigation

At the end of the adsorption process, the spent p(AA-co-MMA) and CMC/p(AA-co-MMA) samples were collected and then soaked in 250 ml stoppered conical flask containing 50 ml ethanol to encourage desorption of safranine dye. The produced suspensions were kept in an orbital shaker at 200 rpm for a period of 6 h. Afterwards, the regenerated polymer samples were centrifuged, washed thrice with deionized water, and conducted in a subsequent adsorption experiment for removal of safranin dye (120 mg/L) from aqueous solution, keeping the other conditions like those described in “Isotherm study” section. The regeneration-reusing process was repeated for another three times, and the removal efficiency (R, %) of safranin dye over the understudied polymeric samples was detected after each run. Each adsorption experiment was performed in triplicate, and the average value was reported linked with calculating ± SD.

### Antimicrobial studies of silver-containing polymeric systems under investigation

Disc diffusion method was used to investigate the antimicrobial activity of Ag@p(AA-co-MMA) and Ag@CMC/p(AA-co-MMA) composites against pathogenic microbes (*Escherichia coli* NCTC10418, and *Staphylococcus aureus* ATCC6538). Firstly, agar was prepared by dissolving the agar powder in water at 4% (w/v) via stirring at 100 °C until a clear light brown color solution was obtained and autoclaved. Then after, the agar was cooled and poured into Petri dishes until it solidified completely. Then bacterial suspension was disseminated onto the surface of solidified agar and incubated for 24 h at 37 °C. Alternatively, the understudied composites were compacted (0.04 g of samples, 6 mm in diameter, pressed by 9.8 MPa 30 s) and incubated for 24 h at 37 °C. Once bacteria were grown in the agar petri dish, the compacted composites were then placed onto the bacterial colony and incubated for another 24 h at 37 °C. After incubation, the zone of inhibition was measured (in mm). The percentage of bacteria reduction (R_B_, %) was calculated using Eq. (13).

13 $${\text{R}}=\left( {{\text{b}}/{\text{a}}} \right) \cdot 100$$

where a and b were inhibition zones for standard antibiotics and examined composite, respectively. Tetracycline 30 and Amoxicillin *AX*-*10* were used as standard antibiotics for *E. coli* and *S. aureus*, respectively. Abbreviations of all terms used in this study were listed in Table S1, Supplementary Information.

## Results and discussions

### Microstructural analyses

**Fig. 1:**
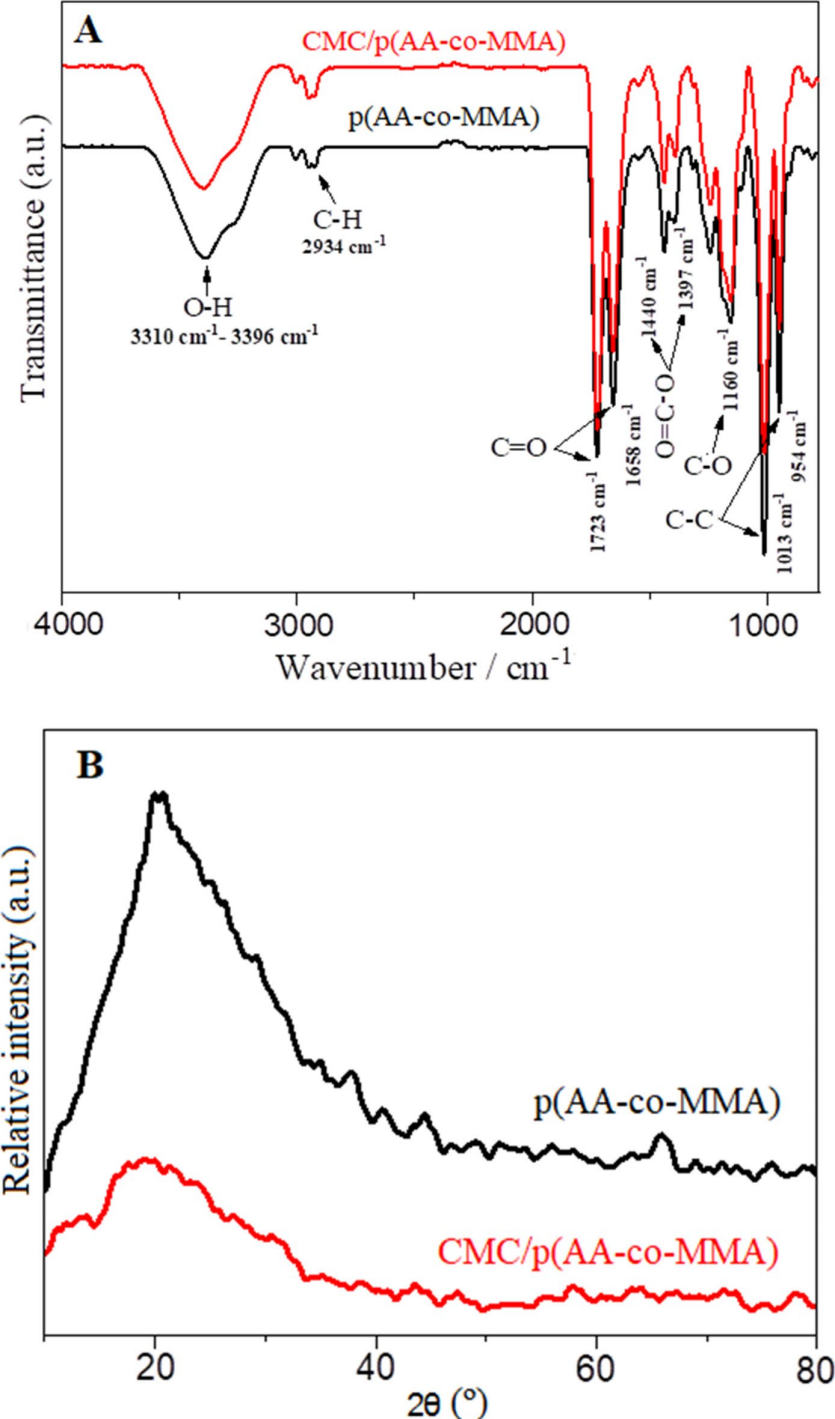
(**A**) FT-IR spectra and (**B**) XRD patterns of p(AA-co-MMA) copolymer and CMC/p(AA-co-MMA) hybrid polymer composite.

FT-IR spectra of p(AA-co-MMA) copolymer and CMC/p(AA-co-MMA) hybrid polymer composite are presented in Fig. 1A. For p(AA-co-MMA) copolymer, a broad band extending from 3310 cm^− 1^ to 3396 cm^− 1^ appears ascribing to O-H stretching vibrations linked with presence of a weak absorption band at 2934 cm^− 1^ referring to C-H stretching vibrations^39^. The sharp and intense peaks at 1723 cm^− 1^ and 1658 cm^− 1^ are assigned to the C = O stretching vibrations^37,40,41^. Two pronounced bands assigned to the symmetrical and asymmetrical (O = C-O) carboxylate ions appeared at 1397 cm^− 1^ and 1440 cm^− 1^, respectively^42,43^. Sharp band characteristic to the stretching vibration of aliphatic ester in the copolymeric chain takes place at 1244 cm^− 1^ as described in the previous report^42^. Moreover, a sharp band at 1160 cm^− 1^ is shown in the FT-IR spectrum of p(AA-co-MMA) sample (Fig. 1A) corresponding to C-O stretching vibration of methyl ester group in copolymeric moiety. In addition, two sharp vibrational bands at 954 cm^− 1^ and 1013 cm^− 1^ appear reflecting presence of skeletal C-C stretch^44^. Upon hybridization of p(AA-co-MMA) with CMC (Fig. 1A), most of these aforementioned peaks are retained with the disappearance of any characteristic peak referring to CMC structure. This result may be attributable to the strong resemblance of the functional groups in carboxymethyl cellulose structure to those found in p(AA-co-MMA) copolymer. Besides, the intactness of FT-IR profile of p(AA-co-MMA) when hybridized with CMC perhaps guarantees the compatibility between carbohydrate and copolymer, where CMC chains seem to intimately interact with the copolymer segments, as being later clarified in this study.

XRD patterns of p(AA-co-MMA) copolymer and CMC/p(AA-co-MMA) hybrid polymer composite are shown in Fig. 1B. It is deduced that the copolymer has a semi-crystalline nature displaying a marked broad and intense peak extending from 2θ = 10° up to ~ 40° complying to previous reports^38–40^. XRD diffractogram of CMC/p(AA-co-MMA) hybrid polymer composite resembles that of p(AA-co-MMA) copolymer but with a remarkable reduction in the intensity of the characteristic peak, Fig. 1B. Such degeneracy in the crystalline nature of p(AA-co-MMA) when hybridized by CMC rises probably from the formation of stable hydrogen bonds between CMC chains and p(AA-co-MMA) copolymer, as being hypothesized in previously reported work^45^. In consequence, CMC is highly encouraged to be tightly attached to the copolymeric matrix, endorsing FT-IR data. As reported in the previous literature, the crystalline nature of carboxymethyl cellulose is significantly enhanced by the formation of intramolecular hydrogen bonding where the hydroxyl groups at C-6 are potentially engaged in this interaction^45,46^. The very same trend, given increasing crystallinity by promotion of intramolecular hydrogen bonding, is asserted in vinyl copolymers^45^.

**Fig. 2:**
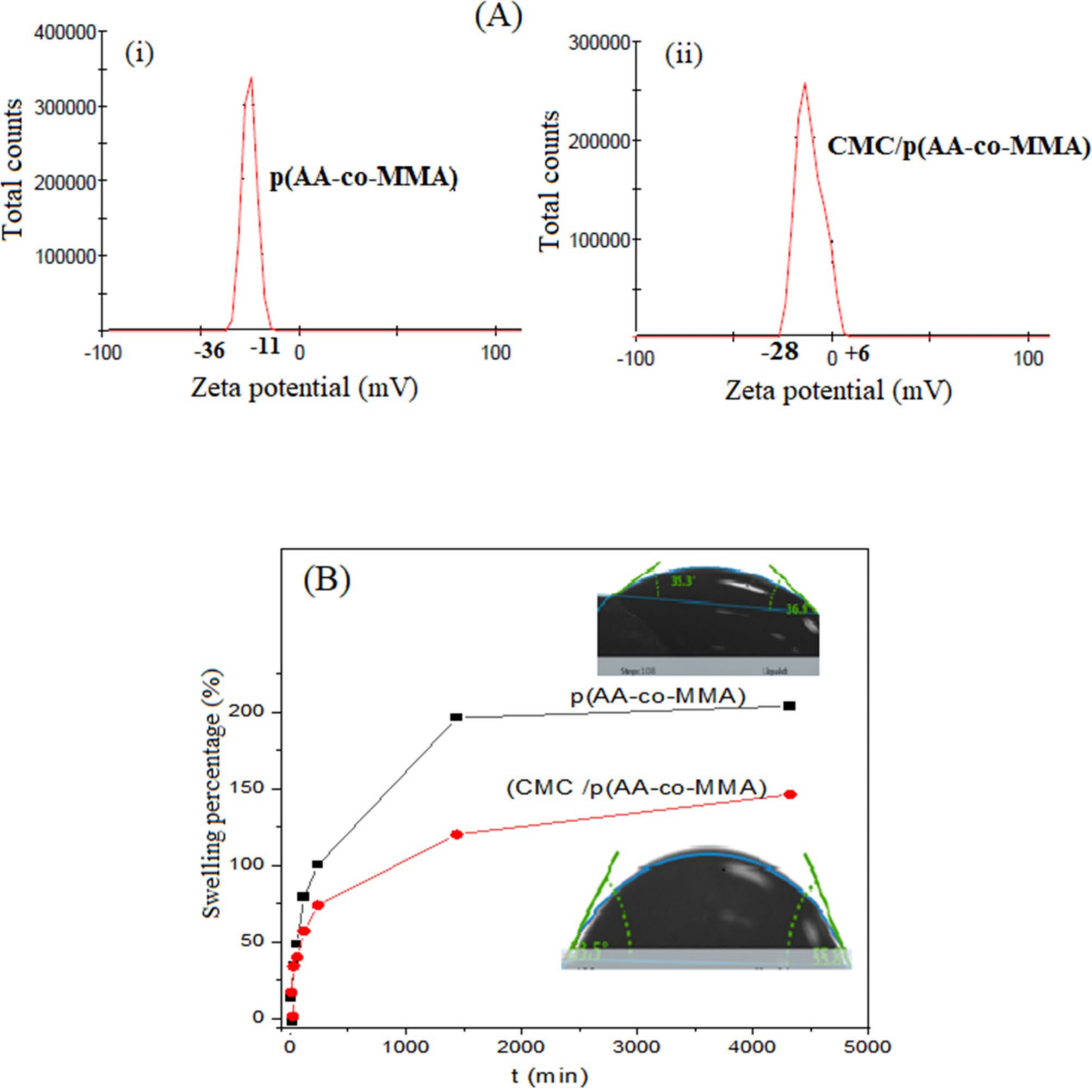
(**A**) Average zeta-potential distribution curves and (**B**) Swelling kinetic curves and contact angle of p(AA-co-MMA) copolymer and CMC/p(AA-co-MMA) hybrid polymer composite.

As plotted in Fig. 2A (i), the zeta potential distribution curve (ZPD) of p(AA-co-MMA) shows a sharp and intense peak starting from − 36 mv to -11 mv with ζav value close to -25 mV. These results reveal the presence of abundant carboxylate anions in the copolymeric chains of p(AA-co-MMA), agreeing well with previous studies^47,48^. After the copolymer was hybridized with CMC, a significant broadening of the ZPD profile was observed. The profile shifted to higher values within the range of -28 mV to + 6 mV, as shown in Fig. 2A (ii). These findings infer earning of CMC/p(AA-co-MMA) hybrid polymer composite to a few positively charged surface sites. It is worthwhile to note here that ζav value of p(AA-co-MMA) copolymer (-29 mV) is two-fold increased when hybridized with CMC chains (-14 mV), indicating a noticeable deficiency in the amount of COO¯ anions, see Fig. 2A (I, ii). This fact reveals the successful endeavour of CMC chains to tightly interact with p(AA-co-MMA) copolymer segments, thus concealing the nucleophilic character of COO¯ ions and causing a marked reduction in the surface charge nature of CMC/p(AA-co-MMA) - in other words, developing the hydrophobic nature of CMC/p(AA-co-MMA). Such acquired respective results concur with FT-IR and XRD results.

### Swelling and contact angle studies

Swelling kinetic curves of p(AA-co-MMA) and CMC/p(AA-co-MMA) are illustrated in Fig. 2B. It is noticeable that hybridization of p(AA-co-MMA) copolymer with CMC leads to considerable deterioration in the swelling rate as well as the equilibrium swelling percentage. The equilibrium swelling percentage of p(AA-co-MMA) copolymer (of ~ 210%) is decreased to half its value when hybridized with CMC chains, Fig. 2B. This phenomenon may strongly reflect the affinity of hydroxyl groups of CMC to aggressively interact with carboxylate anions of p(AA-co-MMA), resulting in a substantial reduction in the hydrophilic nature of copolymeric chains, as being evidenced by DLS study. For more confirmation, the contact angle between water drops and the polymeric surfaces of the as-prepared samples is measured at a proper exposure period (~ 30 s), see inset images of Fig. 2B. It is generally discernible that CMC/p(AA-co-MMA) exhibits a higher contact angle of ca. 55° than that of p(AA-co-MMA) copolymer, which registers a value approaching 32°. These facts reveal the development of the hydrophobic character of CMC/p(AA-co-MMA) compared to that of p(AA-co-MMA), thus presumably confirming the preferential interaction between -OH groups of CMC chains and carboxylic species of p(AA-co-MMA) copolymer rather than being attached to the water drop. Such consensus is a coincidence with the swelling data.

### Morphological analysis

**Fig. 3:**
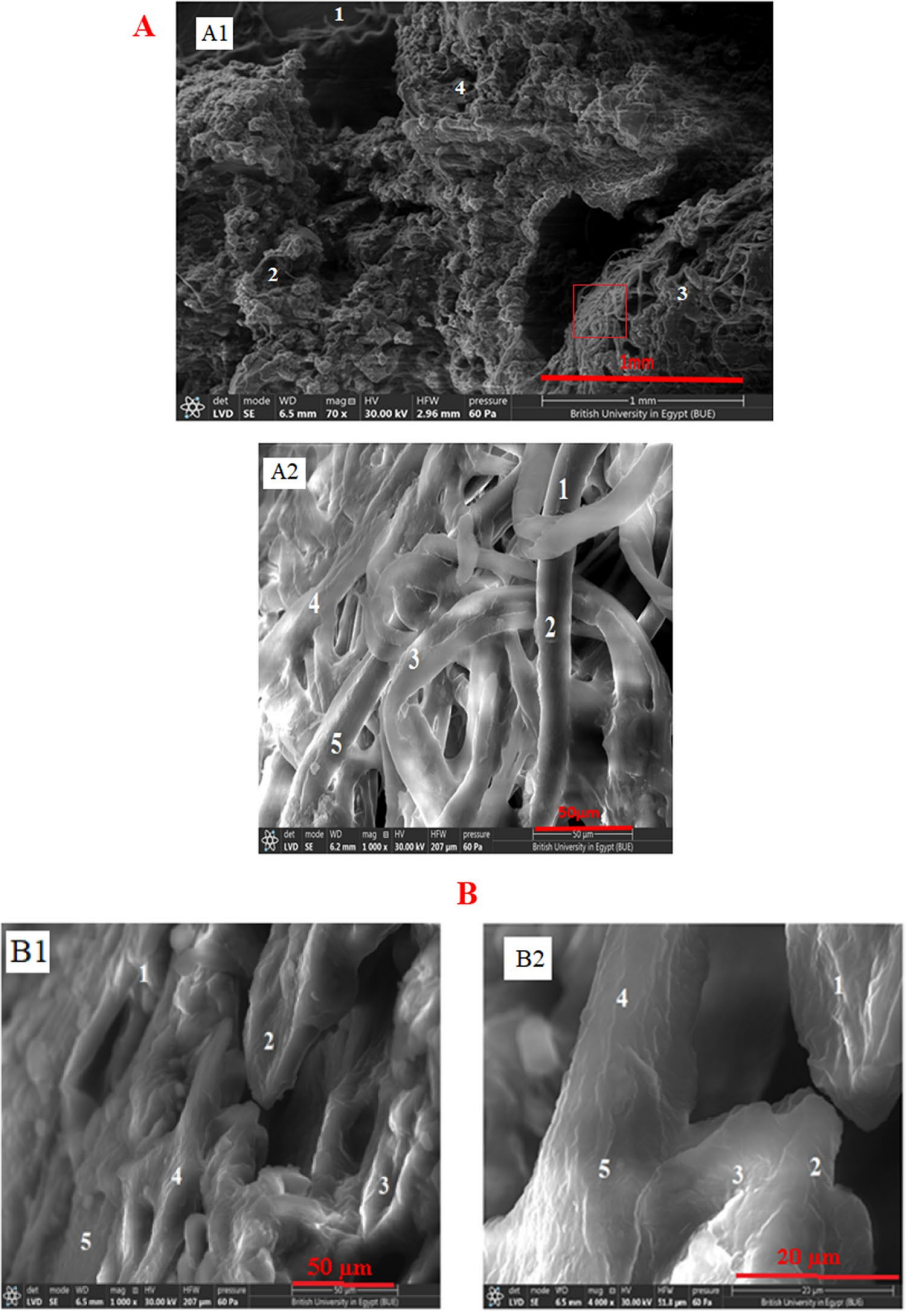
SEM images of: (**A**) p(AA-co-MMA) copolymer and (**B**) CMC/p(AA-co-MMA) hybrid polymer composite.

The morphology of p(AA-co-MMA) copolymer and CMC/p(AA-co-MMA) hybrid polymer composite is analyzed by SEM, Fig. 3A,B. As shown in Fig. 3A1, p(AA-co-MMA) copolymer displays a roughened surface enriched with tangled, zigzagged and interconnected filaments, as represented by elements 1–4. To be more convenient with this vision, the zone in the red box is magnified and presented in Fig. 3A2. It is worth to hypothesize that p(AA-co-MMA) is composed primarily of extended thick filaments with strictly uniform dimensions of about 12 μm in diameter, being folded over each other (Fig. 3A2). In a reverse trend, hybridization of p(AA-co-MMA) with CMC inspires the existence of smooth and homogeneous surfaces of plate-like morphology, as represented by elements 1–5, see Fig. 3B1. A deep look at a higher magnification image (Fig. 3B2) reveals the accessibility of CMC chains to firmly join the p(AA-co-MMA) filaments together forming condensed platy surfaces, as distinguished from elements 1–5. These findings strongly reflect the avidity of CMC to reformulate the p(AA-co-AM) copolymeric filaments endowing them with advanced orientation form. This perception is coherent with the XRD, DLS and contact angle data, which corroborate the intimate interaction of CMC chains to p(AA-co-AM), predominantly proceeding hydrogen bonding.

**Fig. 4:**
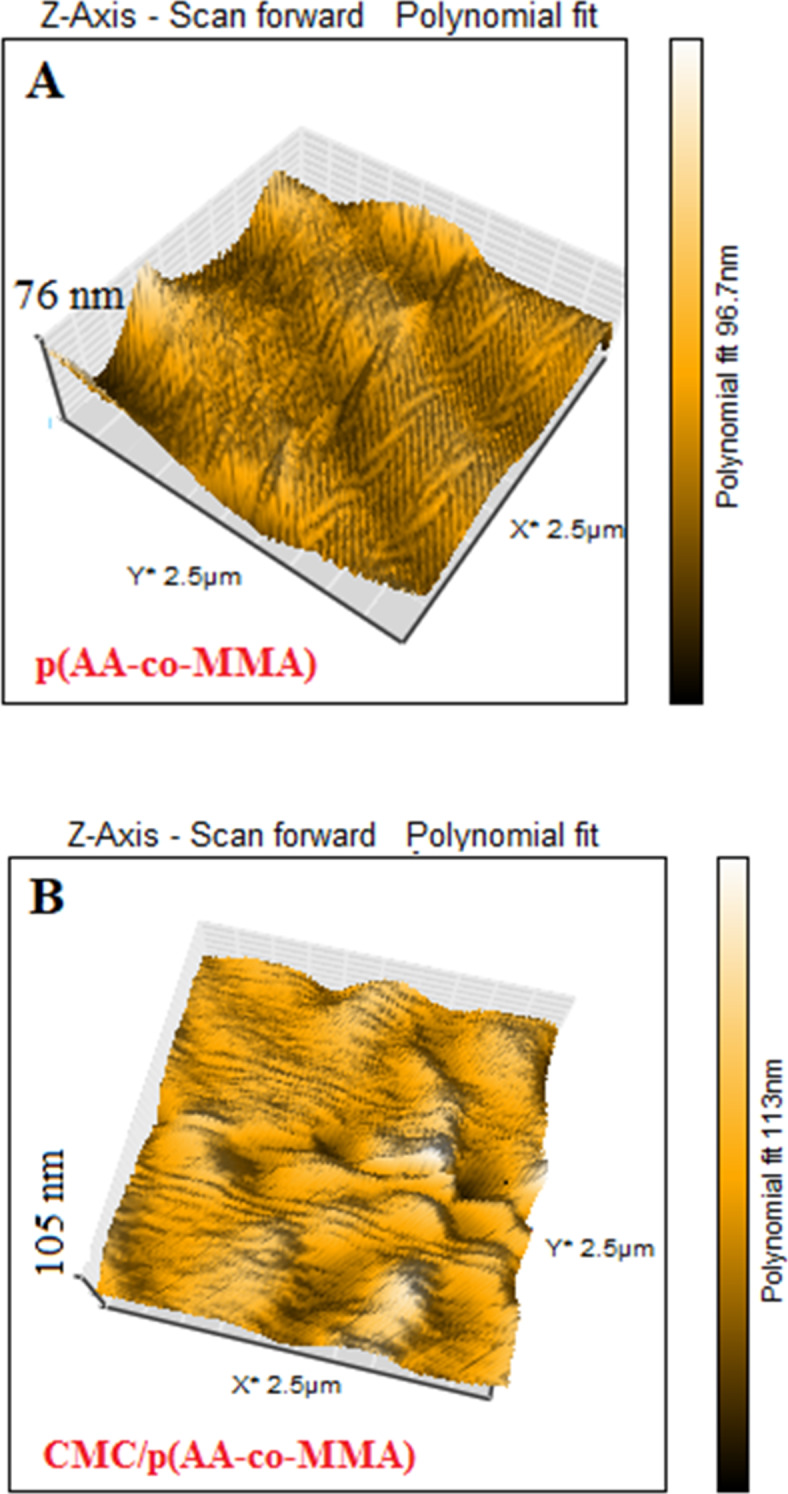
The AFM images of: (**A**) p(AA-co-MMA) copolymer and (**B**) CMC/p(AA-co-MMA) hybrid polymer composite.

The textural characteristics of p(AA-co-MMA) copolymer and CMC/p(AA-co-MMA) hybrid polymer composite are elucidated using AFM analysis, Fig. 4A,B. It is generally acceptable that p(AA-co-MMA) filamentous copolymer seemingly massed to form a dune which appears as a zigzag surface with a significantly roughened nature, cf. Fig. 4A. The height in z-dimension, for p(AA-co-MMA), achieves 76 nm (Fig. 4A). On contrary, for CMC/p)AA-co-MMA), the surface roughness is distinguishably alleviated resulting in presence of a lower zigzag surface containing hill and valley zones linked with heights in z-dimension of 105 nm on average (Fig. 4B) as compared with p(AA-co-MMA) sample. Such remarkable change in the surface topography of p(AA-co-MMA) when hybridized with CMC presumably strives from the successful interconnectedness of the carbohydrate chains to the copolymeric matrix facilitating the formation of intermolecular hydrogen bonding hydrogen, thus endorsing XRD, DLS, contact angle and SEM results.

**Fig. 5:**
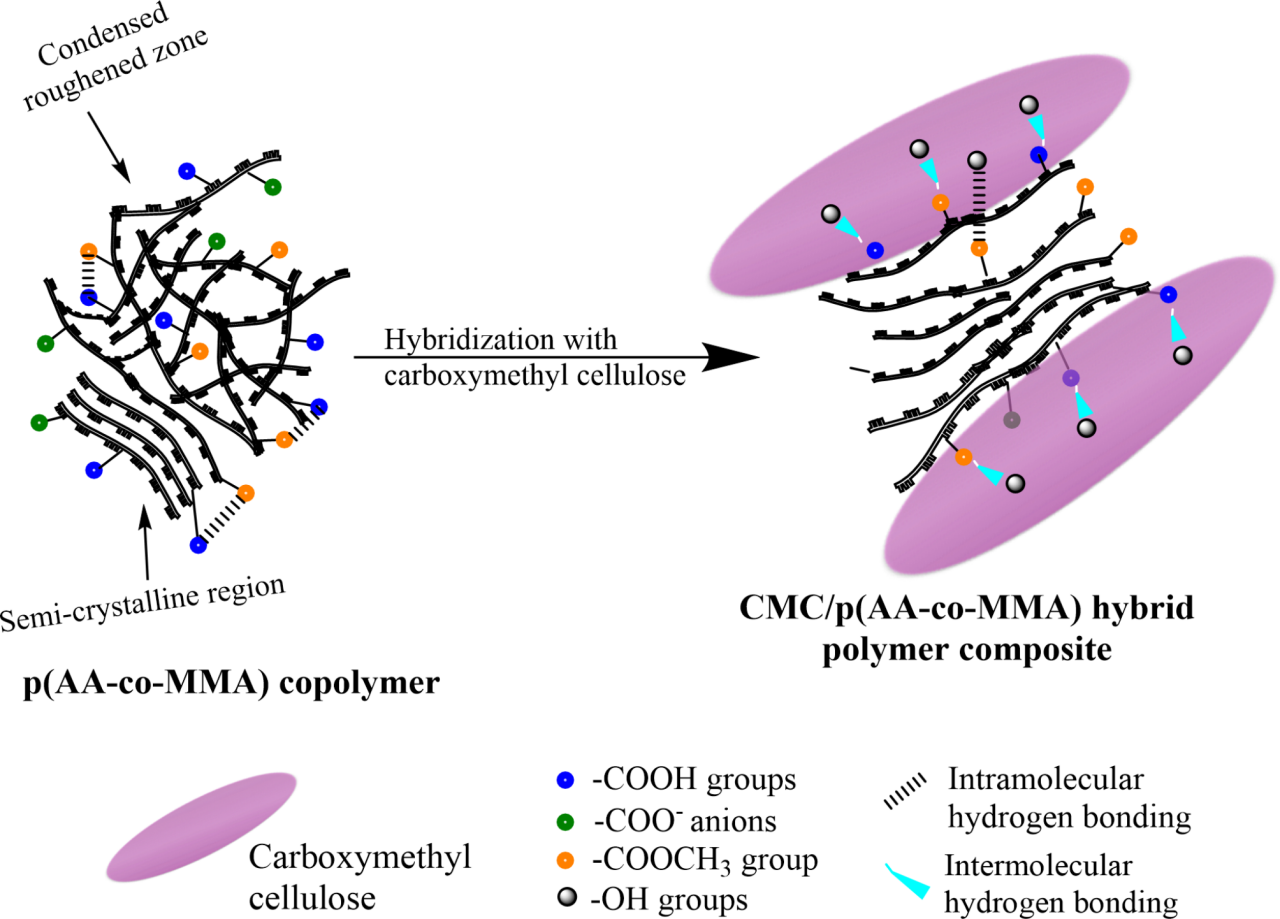
Portrayal view representing the changes in the interaction characteristics of p(AA-co-MMA) copolymer when hybridized with CMC chains.

By considering all the above-mentioned physicochemical properties, it appears tempting to ascertain a representative view for the prospected interaction characteristics in either p(AA-co-MMA) copolymer or its hybridized form with CMC, as shown in Fig. 5. Copolymerization of AA and MMA monomers generates extended folded filaments, which are condensed in some regions and separated in other ones, Fig. 5. Such orientational conformation relies radically on the nature of functional groups attached to the copolymeric segments. The zones that are intensively folded on each other exhibit some crystallinity, which may strongly result from the intramolecular hydrogen bonding between carboxylic and methyl ester groups (Fig. 5), as evidenced by XRD and SEM results. Meanwhile, the areas holding separated filaments are most probably enriched by carboxylate anions originating repulsion between polymeric segments to attain further stability, as verified by DLS and contact angle studies, cf. Figure 5. On the other hand, hybridization of p(AA-co-MMA) with CMC endorses preferential linkage of the hydroxyl groups of CMC to the carboxylic as well as methyl ester groups of p(AA-co-MMA) copolymer through adoption of intermolecular hydrogen bonding, Fig. 5. Furthermore, -OH groups of CMC may interact with carboxylate anions of p(AA-co-MMA) displaying ion-dipole interactions, Fig. 5. Such diverse interacting modes involving CMC structure and p(AA-co-MMA) copolymer plausibly encourage intensive coupling of carbohydrate with copolymeric moiety yielding smooth and regular hybrid polymer composite of plate-like architecture (Fig. 5), as evidenced by SEM and AFM investigations. Firm interaction of CMC to p(AA-co-MMA) most probably masks the nucleophilic nature of carboxylate anions constructing a hybrid polymer composite with somewhat hydrophobic character, complying with DLS, swelling and contact angle measurements.

### Applicability of the polymer-based samples under study in environmental remediation

#### Adsorption performance of p(AA-co-MMA) copolymer and CMC/p(AA-co-MMA) hybrid polymer composite for treatment of dyeing wastewater

This part deals with interpreting the role of CMC in developing the adsorbing behaviour of p(AA-co-MMA) copolymer toward the removal of cationic dyes, namely, safranin dye, from wastewater.

*Adsorption equilibrium studies*

**Fig. 6:**
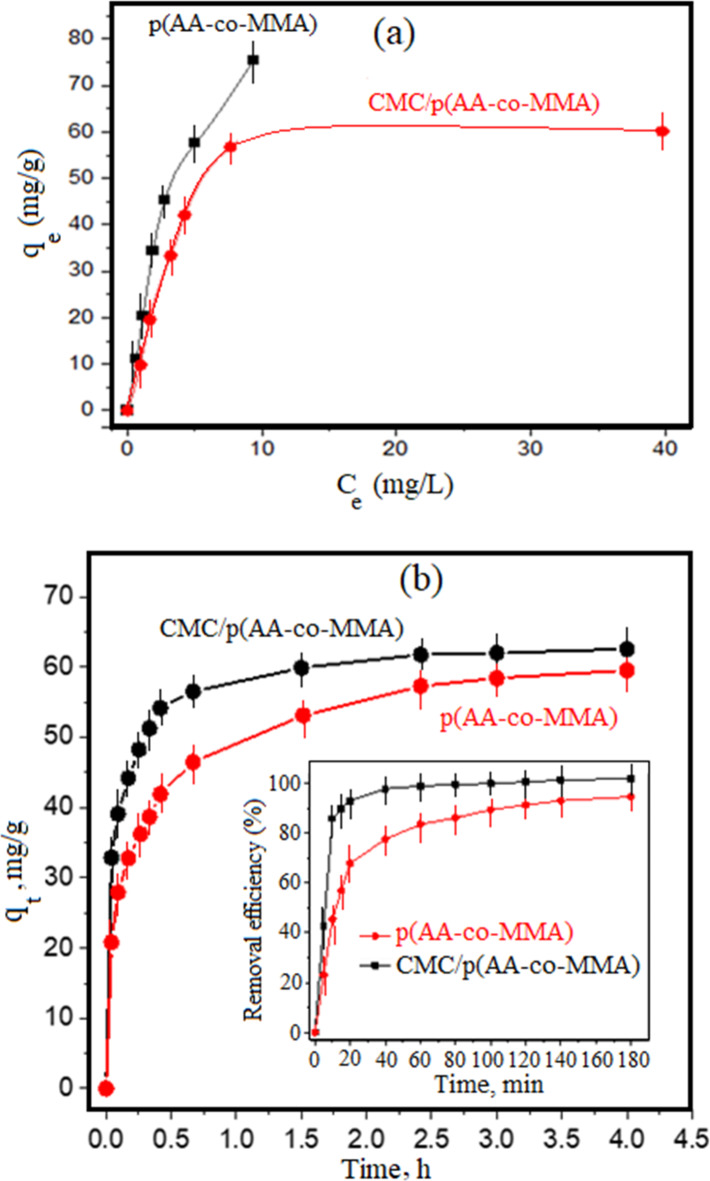
Equilibrium adsorption isotherms (**a**) for removal of safranin dye over p(AA-co-MMA) copolymer and CMC/p(AA-co-MMA) hybrid polymer composite (pH: 7.8; sorbent dosage: 2 g/L; initial dye concentrations: 10, 20, 40, 90, 120, 160 mg/L; temperature 25°C; contact time: 3 h), and kinetic study (**b**) for adsorption of safranine dye over p(AA-co-MMA) copolymer and CMC/p(AA-co-MMA) hybrid polymer composite (pH 7.8; sorbent dosage: 2 g/ L; initial dye concentration: 120 mg/L; temperature 25°C).

The adsorption equilibrium studies of p(AA-co-MMA) copolymer and CMC/p(AA-co-MMA) hybrid polymer composite are performed and presented in Fig. 6a. For p(AA-co-MMA), the adsorption capacity of safranin dye is linearly increased as a function of the equilibrium concentration, whereas the saturation level is seemingly hard to achieve. On the other hand, a sharp increase in the amount of safranin dye adsorbed over CMC/p(AA-co-MMA) is observed with increasing the equilibrium concentration accompanied by the presence of plateau, Fig. 6a. These findings infer the capability of CMC chains in the hybrid polymer composite to limit the adsorption behaviour of p(AA-co-MMA), thus facilitates from achieving the saturation adsorption capacity. Such limitation is perhaps esteemed from the hydrophobic nature of CMC/p(AA-co-MMA), which is earned by masking the negative charges of carboxylate anions during hybridization process, as evidenced by DLS, swelling and contact angle studies. The adsorption equilibrium curves of both p(AA-co-MMA) and CMC/p(AA-co-MMA) are examined by applying Freundlich, Langmuir, and Dubinin–Radushkevich isotherm models. The derived adsorption parameters from these models are scheduled in Table 1 and SI (Fig. S1). It is acclaimed that p(AA-co-MMA) copolymer follows Freundlish and Langmuir approaches with a coefficient of determination close to 0.99, reflecting presence of homogeneous surfaces side by side with heterogeneous ones. This copolymer exhibits promising adsorption avidity to cationic dye yielding Freundlich and Langmuir coefficient values of 2.05 mg^1 − 1/n^ L^1/n^ g^− 1^ and 0.0017 L mg^− 1^, respectively, being much higher than those prescribed in previous literature reports^49,50^. Alternatively, Langmuir adsorption progression is only fitted for CMC/p(AA-co-MMA) hybrid polymer composite with a coefficient of determination (r^2^) close to unity, authenticating presence of regular and homogeneous surface, being consistent with SEM and AFM analyses. Compared to p(AA-co-MMA), Langmuir constant value (b, L mg^− 1^) of CMC/p(AA-co-MMA) is nearly doubled, thus alleging the effectiveness of the sole contribution of surface homogeneity in developing the adsorption nature of the hybrid composite under study, see Table 1. In addition, 0 < R_L_<1 implies that the adsorption process is considerably favoured. Turning to the outstanding willingness of dye adsorption onto the as-prepared copolymer, Fig. 6a, D-R approach is not well-fitted for p(AA-co-MMA) sample owing to the failure to attain saturation adsorption capacity. This fact presumably accentuates the facilitated interaction between the dye molecules and the copolymeric matrix, where columbic and ion-dipole forces and ion pairing seem to work collaboratively during the adsorption of safranine onto p(AA-co-MMA) copolymer. Controversely, the Dubinin-Radushkevich (D-R) approach is significantly valid for dye adsorption over CMC/p(AA-co-MMA) sample (r^2^ = 0.97) and records a saturation adsorption capacity value of ca. 59.47 mg g^− 1^, being quite relevant to that distinguished from its corresponding adsorption equilibrium isotherm, Table 1. In addition, CMC/p(AA-co-MMA) exhibits apparent adsorption-free energy around 1.3 kJ/mol (Table 1), being much lower than those reported in previous studies^1,49–51^. These results affirm that hybridization of p(AA-co-MMA) with CMC plausibly weakens the interaction between safranin dye and copolymeric moiety, judiciously supposing the engagement of hydrogen bonding and/or dispersion forces in the adsorption progression.

**Table 1:** Freundlich, Langmuir and Dubinin–Radushkevich coefficients for safranin adsorption at 25°C (pH: 7.8; sorbent dose: 2 g/ L; initial dye concentrations: 10, 20, 40, 90, 120 and 160 mg/L; contact time: 3 h).

Sorbents	Freundlich model		Langmuir model			Dubinin-Radushkevich model		
	K_F_ (mg^1-1/n^ L^1/n^ g^-1^)	r^2^	K_L_ x10^2^ (L mg^-1^)	r^2^	R_L_	q_D_ (mg g^-1^)	E (kJ mol^-1^)	r^2^
p(AA-co-MMA)	2.05	0.95	0.17	0.93	0< R_L_ <1	unreal value	unreal value	0.76
CMC/p(AA-co-MMA)	not determined	0.75	0.29	0.99	0< RL <1	59.47	1.36	0.97

*Kinetic study*

The adsorption kinetics of safranin dyes over p(AA-co-MMA) and CMC/p(AA-co-MMA) hybrid polymer composite are represented in Fig. 6b. It can be deduced that CMC/p(AA-co-MMA) possesses elevated adsorption rates rather than those of p(AA-co-MMA), whereas the hybrid polymer composite records adsorption capacity close to 55 mg/g within 25 min while the understudied copolymer shows adsorption uptake around 40 mg/g for the same defined adsorption period, Fig. 6b. However, the maximum adsorption capacity of CMC/p(AA-co-MMA) hybrid composite is nearly comparable to that of p(AA-co-MMA) showing a value in the vicinity of 60 mg/g. As visualized in the inset of Fig. 6b., the removal efficiencies of CMC/p(AA-co-MMA) are drastically increased with time displaying values greater than those of p(AA-co-MMA) copolymer. Near 90% removal efficiency of safranine dye from wastewater is achieved after approximately 10 min exposure to CMC/p(AA-co-MMA), while similar contact time is required for just 50% dye removal efficiency, cf. the inset of Fig. 6b. The kinetic parameters, including, pseudo-first order, pseudo-second order, and intraparticle diffusion model are presented in Table 2 and SI (Fig. S2). It is pertinent to mention that both samples under study follow pseudo-first- and second-order models with a coefficient of determination close to unity revealing the presence of various adsorption profiles during the removal of safranin from aqueous solution. These adsorption profiles may depend on the concentration of safranin dye in wastewater and/or the interacting modes gathering the dye molecules with the sorbent materials. Of special attention, specific rate constants of CMC/p(AA-co-MMA) hybrid polymer composite are much higher than those of p(AA-co-MMA), Table 2, confirming the beneficial contribution of hydroxyl groups of carboxymethyl cellulose in developing a great deal of hydrogen bonds with dye molecules, thus, in turn, accelerating the adsorption rates for removal of safranin from wastewater. Besides, the relative hydrophobicity acquired in CMC/p(AA-co-MMA), as confirmed by DLS analysis, perhaps be an additional cause for the presence of dispersion forces between dye molecules and depolluting material. Such prospective comportments are much more pronounced by applying the second order approach, where the specific rate constant of CMC/p(AA-co-MMA) is one and half-fold increasing that of p(AA-co-MMA). By applying the intraparticle diffusion model, CMC/p(AA-co-MMA) hybrid polymer composite serves a highly developed external mass transfer rate constant (k_i_^I^) rather than that obtained by p(AA-co-MMA) copolymer, see Table 2. In a reverse trend, the internal diffusion rate constant of dye molecules within the pore channels of CMC/p(AA-co-MMA) is notably diminished when compared with that derived from dye adsorption over p(AA-co-MMA), i.e., the pore diffusion rate constant (k_i_^F^) of CMC/p(AA-co-MMA) is two-fold lower than that recorded by p(AA-co-MMA), Table 2. These findings plausibly present the great affinity of CMC chains to efficiently bind with the copolymer matrix resulting in blockage of the copolymeric voids, a fact that is guaranteed by owing of CMC/p(AA-co-MMA) to smooth surface of plate-like morphology (as evidenced by SEM and AFM analyses). Such morphological attitude facilitates the diffusion of dye molecules to the surface of the hybrid polymer composite and places constraints on the diffusion of dye molecules inside the polymer porous structure. In an alternative manner, the filament-like structure in p(AA-co-MMA) endows the copolymer with a significant porous structure that encourages internal diffusion of dye molecules within the copolymer pore network rather than being externally diffused over the roughened copolymeric surface. These outputs are in line with those obtained from the adsorption isotherm study.

**Table 2:** Pseudo-first order and pseudo-second order kinetics, and intraparticle diffusion model for safranin removal from wastewater using various adsorption systems under study at 25°C (pH 7.8; sorbent dose: 2 g/L; initial dye concentration: 120 mg/L).

Sorbents	Pseudo-first order model		Pseudo-second order model		Intra-particle diffusion model		
	k_1_ × 10^2^ (h^-1^)	r^2^	k_2_ × 10^2^ (g mg^-1^ h^-1^)	r^2^	k_i_^I^ x 10^2^ (mg g^-1^ h^-0.5^)	k_i_^F^ x 10^2^ (mg g^-1^ h^-0.5^)	r^2^
p(AA-co-MMA)	86	0.98	10	0.99	37.4	10.2	0.99
CMC/p(AA-co-MMA)	106	0.97	15	0.98	48.8	5.3	0.98

*Thermodynamic study*

It is imperative to study the thermodynamics of safranin adsorption over p(AA-co-MMA) and CMC/p(AA-co-MMA) samples to interpret the adsorption mechanism. The thermodynamic plot is depicted in SI (Fig. S3), and the thermodynamic parameters are presented in Table 3, including, enthalpy (∆H°), entropy (∆S°), and Gibbs free energy (∆G°). As shown in Table 3, the p(AA-co-MMA) and CMC/p(AA-co-MMA) hybrid polymer exhibit negative ∆H° and ∆S° values inferring exothermicity of the adsorption process with a reasonable ordering profile. Of special interest, all the computed ∆H° and ∆S° values never exceed 65 kJ mol^− 1^ pointing most probably to the sole participation of physical forces in the adsorption process^1,52^. Interestingly, the p(AA-co-MMA) copolymer shows developed negative enthalpy and entropy values of approximately 1.5 times greater than those recorded by CMC/p(AA-co-MMA), Table 3. These findings strongly reflect the presence of electrostatic forces between the carboxylate anions in the copolymeric segments of p(AA-co-MMA) and the nitrogen cationic centres of safranin dye. Enriching of p(AA-co-MMA) copolymer with carboxylate anions is formerly evidenced by zeta-potential analysis. Furthermore, the carboxylic and methyl ester groups in the copolymeric moiety are favourably hydrogen bonded with amine groups of safranine molecules. On the other hand, the reduced values of ∆H° and ∆S° for CMC/p(AA-co-MMA) hybrid polymer composite imply the existence of adequate interactions between dye molecules and hybrid polymer composite, which possesses hydrophilic/hydrophobic nature, that serve hydrogen bonding and dispersion forces, being quite familiar with DLS, and adsorption isotherm and kinetic studies. Such expedient interaction may be strongly advantageous for reusability study as discussed later. Moreover, for p(AA-co-MMA) and CMC/p(AA-co-MMA) samples, the growing negative values of ∆G° with increasing temperature assume that adsorption of safranine molecules, which are governed by physical forces, becomes more spontaneous and favourable at higher temperatures^1,49^.

**Table 3:** Thermodynamic parameters of safranin dye removal using the adsorption systems under study at 25°C, 35°C and 50°C. Conditions: pH 7.8; sorbent dose: 2 g/L; initial dye concentration 120 mg/ L; equilibrium time 3 h.

Adsorbents	T (K)	∆G° (kJ mol ^⁻1^)	∆H°( kJ mol ^⁻1^)	∆S° (kJ k^⁻1^ mol^-1^)
p(AA-co-MMA)	298	-6.23	-61.15	-0.18
	313	-1.13		
	323	-10.53		
CMC/p(AA-co-MMA)	298	-6.53	-48.24	-0.11
	313	-4.94		
	323	-7.70		

*Reusability studies*

**Fig. 7:**
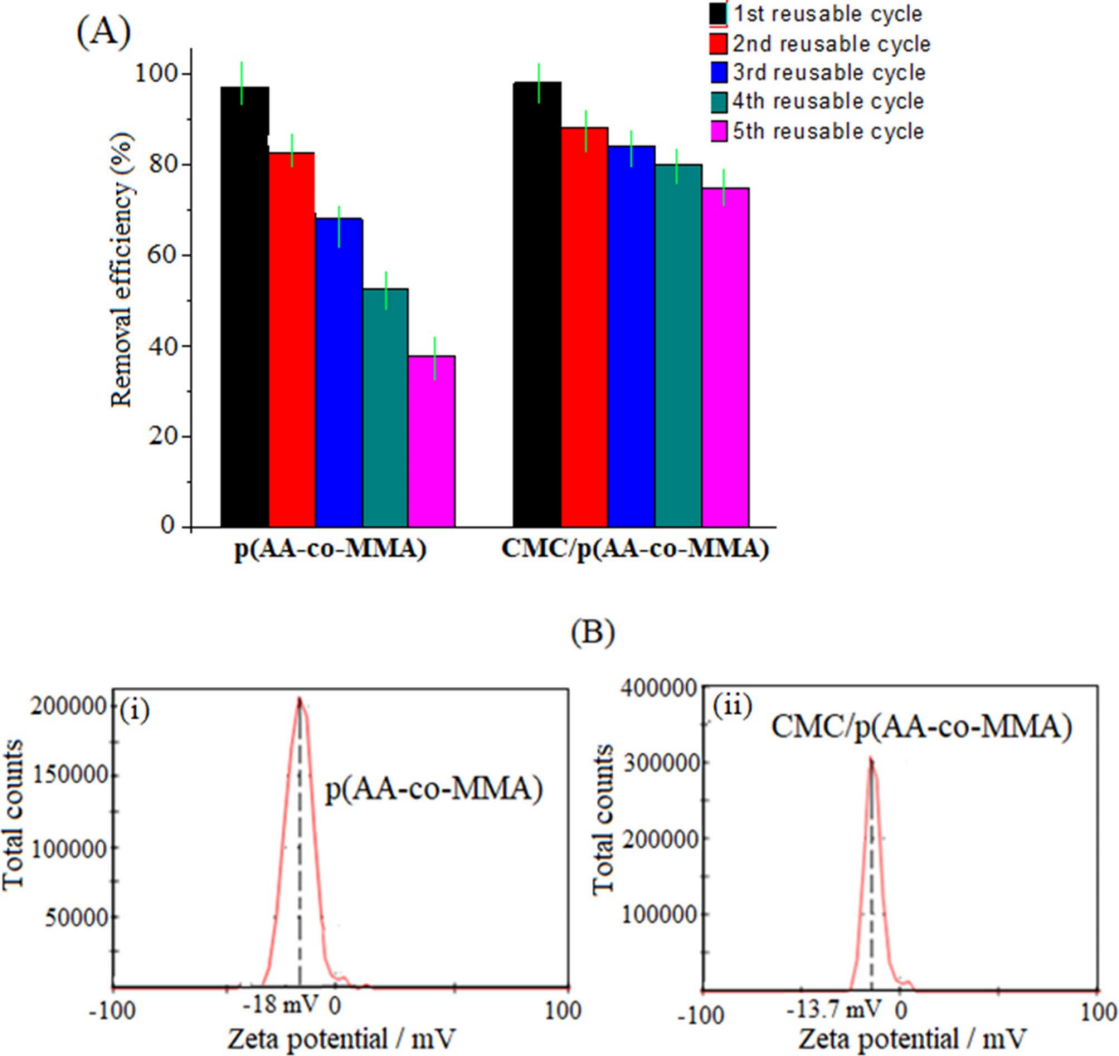
(**A**) Reusability study of p(AA-co-MMA) copolymer and CMC/p(AA-co-MMA) hybrid polymer composite (Set-up conditions: pH: 7.8; sorbent dosage: 2 g/L; initial dye concentrations: 120 mg/L; temperature 25°C; contact time: 3 h), and (**B**) Zeta-potential distribution curve of the exhausted p(AA-co-MMA) and CMC/p(AA-co-MMA) samples after the fifth cycle of dye removal from wastewater.

The reusability of p(AA-co-MMA) and CMC/p(AA-co-MMA) samples towards safranin removal from the aqueous medium is investigated and presented in Fig. 7A. It is worth noting here that the removal efficiency of p(AA-co-MMA) is remarkably deteriorated with multi-usage, recording a removal efficiency below ~ 40% after five consecutive runs (Fig. 7A). Such decline in the removal efficiency of dye may be indicative of the strong electrostatic interaction between safranine molecules and copolymer, thus remarkably obstructs the regeneration process. On the other hand, for CMC/p(AA-co-MMA) hybrid polymer composite, the removal efficiency of safranine dye from wastewater is almost maintained recording values greater than 75% within five reusable cycles, Fig. 7A. This fact reveals the accessibility of the adsorption centres in CMC/p(AA-co-MMA) to efficiently captivate dye molecules and effectively free them up during the regeneration process; so that such conviction is thought to be acceptable when the adsorption progression is dominated by adequate physical forces like hydrogen bonding and dispersion forces, as being demonstrated from adsorption isotherm, kinetics and thermodynamic studies. To gain insight into the enhanced competence and chemical stability of CMC/p(AA-coMMA) hybrid polymer composite over p(AA-co-MMA) copolymer during the conduction of several wastewater purification cycles, the surface charge characteristics of their corresponding exhausted samples are assessed at the end of the fifth purification cycle via detecting their zeta-potential distribution curves, Fig. 7B (i, ii). It is conceivable to hypothesize that multi-usage of p(AA-co-MMA) as an adsorbent for the removal of safranine dye from wastewater distinguishably reduces the negatively charged nature of such copolymer, as the ζ_av_ value is increased from − 25 mV for fresh copolymer to -18 mV for the exhausted one, see Figs. 2A(i) and 7B (i). These results coincide with the adsorption and reusability studies reflecting the failure of regeneration process and the resilient attachment of dye molecules to the adsorbent sites in the copolymer matrix. In the case of CMC/p(AA-coMMA) sample (Fig. 7B, ii), it deserves to be mentioned that ζ_av_ value of the exhausted hybrid polymer composite is intact after being repetitively used in dyeing wastewater treatment for five cycles when compared with that of fresh one (Fig. 2A, ii). This finding may strongly reveal the successfulness of the regeneration-reuse protocol and the ability of CMC chains to create an optimal mode of interaction gathering the dye molecules and the hybrid polymer composite together.

**Fig. 8:**
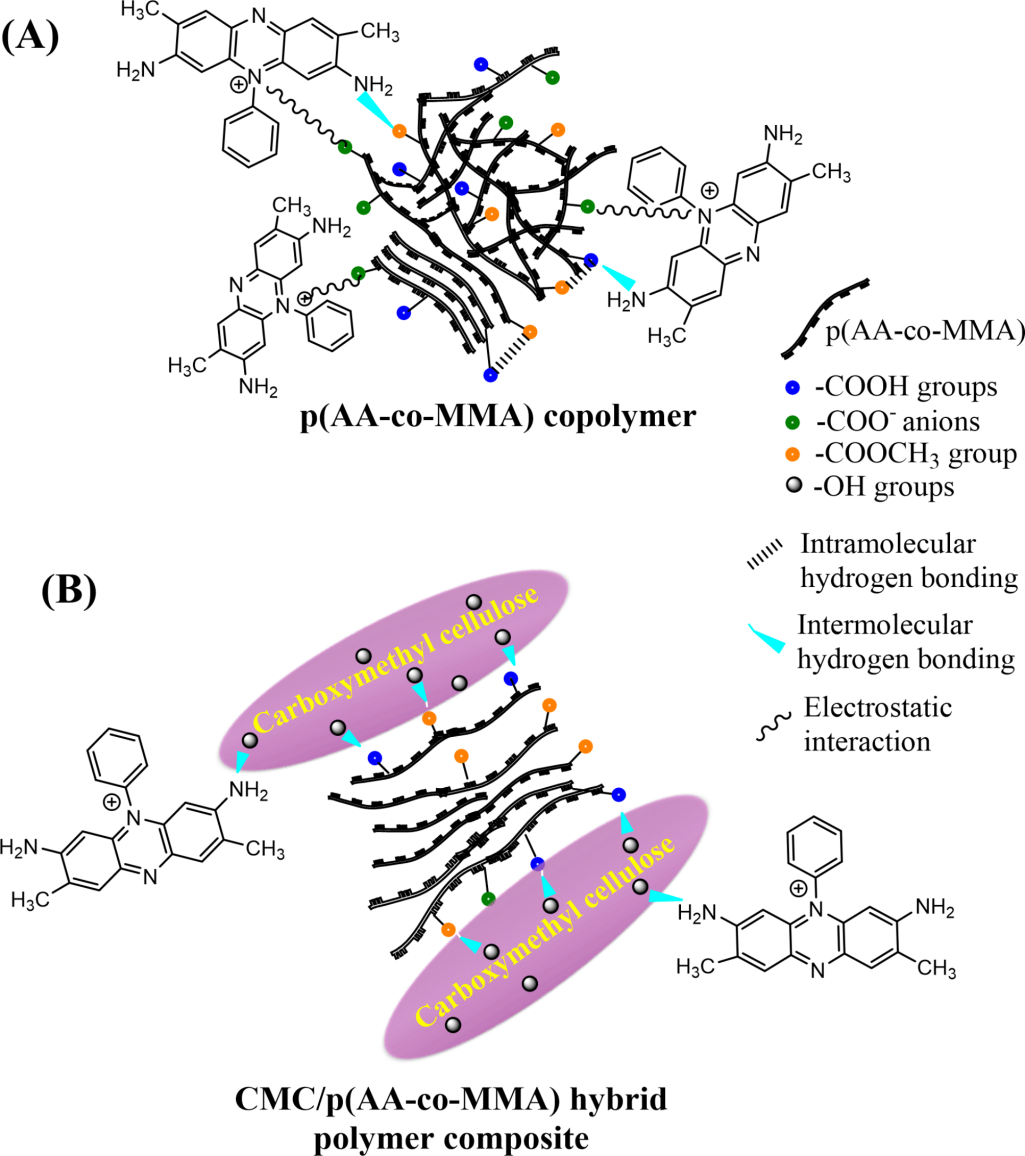
Panoramic view of interaction characteristics adopting the sorption of safranine dye molecules over p(AA-co-MMA) copolymer (**A**) and CMC/p(AA-co-MMA) hybrid polymer composite (**B**).

In light of the above discussion, a panoramic view of the interaction characteristics adopting the sorption of safranine dye molecules over p(AA-co-MMA) and CMC/p(AA-co-MMA) is represented in Fig. 8A,B. It is appreciable to mention that sorption of safranine molecules over p(AA-co-MMA) copolymeric matrix is controlled by the presence of strong physical forces, including, (1) the electrostatic interaction of the positively charged nitrogen centres in safranine dye with carboxylate anions in the copolymeric moiety; (2) the hydrogen bonding between amine groups in safranine molecule, and carboxylic as well as methyl ester groups in the copolymeric filaments, Fig. 8A. However, sorption of dye molecules over CMC/p(AA-co-MMA) may follow different tactics based on implementing balanced interactions of safranine molecules with the hybrid polymer composite matrix. Such physical interactions involve hydrogen bonding between hydroxyl groups of CMC and amine groups of safranine, and dispersion forces between the hydrocarbon chains of dye molecules and depolluting material, see Fig. 8B.

*Comparative study for the removal of safranine dye from wastewater using different depolluting materials*

Adsorption performance parameters, including, adsorbent dosages, removal efficiencies, adsorption rate constants and reaction period, during the decontamination of wastewater from safranine dye using CMC/p(AA-co-MMA) hybrid polymer composite are compared with other previously depolluting systems presented in literature^3,53–58^, Table 4. It is worthwhile to mention that the CMC/p(AA-co-MMA) hybrid polymer composite possesses the highest adsorption efficiency (nearly 100%) and the least adsorbent dosage (2 g/L) whilst safranine dye is rapidly removed from wastewater in a minimal period of ca. 20 min, raising the adsorption rate to the peak (~ 1.06 min^− 1^), see Table 4. These findings stand CMC/p(AA-co-MMA) hybrid polymer composite as a flawless alternative adsorbent compared to the previously presented ones in literature^3,53–58^, see Table 4.

**Table 4:** Comparison of various adsorbent performance parameters perceived from the removal of safranine dye from wastewater using different depolluting materials.

Depolluting materials	Adsorbent dosage, g/L	Removal efficiency, %	Adsorption rate, min^-1^	Adsorption period, min	Reference
Modified Biomaterial - *Bambusa Tulda*	10.0	99.0	0.046	75	54
Poly (anilinecoaniline2,5disulfonic acid)	4.0	73.6	0.109	75	55
Poly (anilinecoaniline2,5disulfonic acid)/ Lhexuronic acid/Ag@SiO2 nanocomposite	4.0	59.3	0.070	75	55
Castor Leaves-based Biochar	5.0	99.6	0.022	90	56
Activated carbon/nano iron oxide	2.5	97.7		120	3
Polystyrene foam	10	96.5	0.0054	60	57
Agar-grafted-graphene oxide	8	80.2	0.025	50	58
Rice husk	1	78.5		60	59
CMC/p(AA-co-MMA) hybrid polymer	2.0	99.2	1.06	40	Present study

#### Microbial disinfection performance of the understudied silver-containing polymer composites in wastewater

**Fig. 9:**
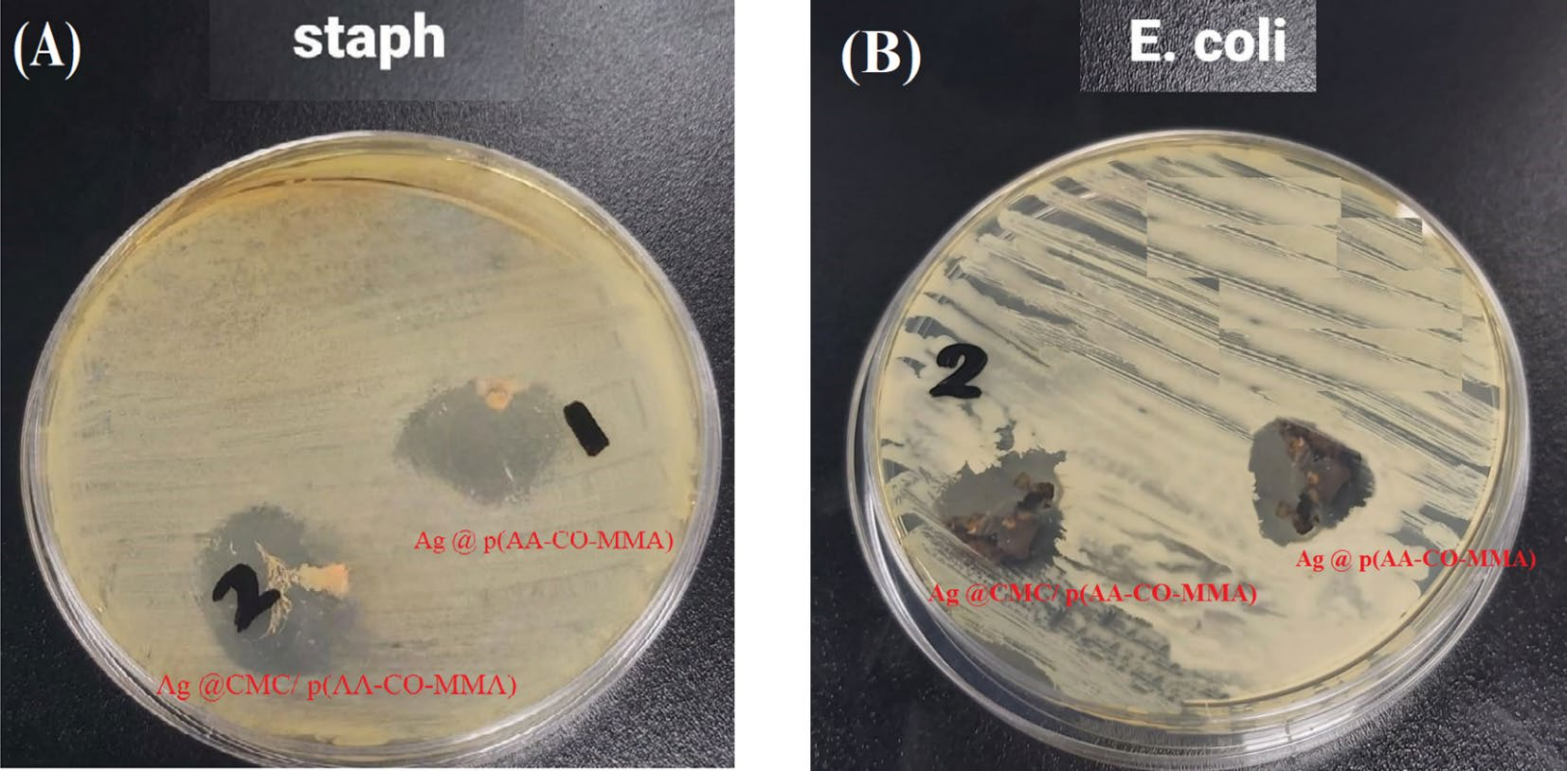
Visual detections of antimicrobial activity of Ag@p(AA-co-MMA) and Ag@CMC/p(AA-co-MMA) composites against different pathogenic microbes: (**A**) *Staphylococcus aureus* ATCC6538 and (**B**) *Escherichia coli* NCTC10418.

The antimicrobial performance of silver embedded onto p(AA-co-MMA) and CMC/p(AA-co-MMA) is investigated by recording the inhibition zones (DIZ) and the microbial reduction percentage (R_B_, %), Table 5. As shown in Table 5, Ag@CMC/p(AA-co-MMA) composite exhibits superior biocidal efficacy to the other one. Extraordinarily, this composite possesses escalated biocidal activity recording two-fold increasing efficiency against *S. aureus* in comparison to that of reference antibiotic (Amoxicillin AX-10), Table 5. In addition, the antimicrobial performance of Ag@CMC/p(AA-co-MMA) composite toward *E. coli* is significantly developed exceeding the microbial reduction of Ag@p(AA-co-MMA) composite by about 25%, Table 5. Furthermore, Fig. 9A,B represents the macro-images of the antimicrobial activity of the understudied composites against *S. aureus* and *E. Coli* bacterial colonies demonstrating the superiority of the exterminated action of Ag@CMC/p(AA-co-MMA) composite over that of Ag@p(AA-co-MMA). Based on literature reports^59–64^, a comparison of the antimicrobial performances (DIZ) against *S. aureus* and *E. Coli* using different biocidal composites is carried out and tabulated in Table 6. It is generally accepted that Ag@CMC/p(AA-co-MMA) composite is competent to be one of the pioneer disinfecting agents for the removal of Gram (+) and Gram (-) bacteria from wastewater. According to the reported literature^59–64^, the stunning biological activity of Ag@CMC/p(AA-co-MMA) composite stems most probably from (i) the developed controlled-release of Ag particles from CMC/p(AA-co-MMA) hybrid polymer moiety, which contains convenient hydrophobic/hydrophilic character^50^, and (ii) the deep impact of the host material, namely, CMC/p(AA-co-MMA) hybrid polymer composite, on the redox potential of Ag particles and their dissolution characteristics for generation of active Ag^+^ ions capable to invade bacterial cell wall and cause degeneration of bacterial metabolism through production of reactive oxygen species^52,65^. In the forthcoming paper, the pharmacokinetic and drug release studies as well as the cytotoxicity of Ag@CMC/p(AA-co-MMA) composite will be scrutinized.

**Table 5:** Antimicrobial activities of p(AA-co-MMA) and CMC/p(AA-co-MMA) against *E. Coli* and *S. Aureus*, compared with biocidal activity of the standard antibiotics against these microbes.

Samples	E. coli		S. aureus	
	DIZ, mm	^R^_B_, ^%^	DIZ, mm	^R^_B_^, %^
Tetracycline 30	20	100	---	---
Amoxicillin AX-10	---	---	10	100
Ag@p(AA-co-MMA)	10	50	19	98
Ag@CMC/p(AA-co-MMA)	15	75	20	200

**Table 6:** Comparison of the antimicrobial performance, in terms of inhibition zone, toward *E. Coli* and *S. aureus* using different silver-containing biocidal composites.

Biocides nanocomposites	Zone of inhibition for E. coli, mm	Zone of inhibition for S. aureus, mm	References
Ag@poly (vinyl alcohol/polyaniline)	12	15	^60^
Ag@Guar gum/curcumin	13	13	^61^
Ag@Poly (vinyl alcohol/karaya gum)	14	14	^62^
Ag@chitosan grafted polyvinyl alcohol	15	-	^63^
Ag@Chitosan	15	-	^64^
Ag@Gum acacia grafted poly methylacrylic acid	16	14	^65^
Ag@Gum acacia grafted poly(methylacrylic acid-co-acrylic acid)	21	16	^65^
Ag@CMC/p(AA-co-MMA)	15	20	Present study

## Conclusions

In this study, authors issued to develop the removal performance and bioactive nature of poly (acrylic acid-co-methylmethacrylate) copolymer, p(AA-co-MMA), via being hybridized with oxygen-rich polymeric chains, in particular, carboxymethyl cellulose (CMC). The physicochemical characteristics of the as-prepared hybrid polymer composite were characterized using XRD, FT-IR, DLS, SEM, and AFM analyses as well as swelling and contact area studies. Hybridization of p(AA-co-MMA) copolymer with CMC chains facilitated development of highly compatible composite, where the carbohydrate molecules were strongly attached to p(AA-co-MMA) filaments via hydrogen bonding, thus imposing the copolymeric fibers to gather and form smooth and homogeneous surfaces of plate-like morphology with poor surface charge characteristics (ζ_Av_ ~ -11 mV, being two-fold higher than that of the copolymer).

Hybridizing of p(AA-co-MMA) copolymer with CMC chains significantly enthused the adsorbing nature of the copolymer toward removal of safranine dye, as a representative cationic dye, from wastewater, yielding near 100% efficiency within not more than 35 min for five successive runs. Adsorption of safranine onto CMC/p(AA-co-MMA) hybrid polymer composite fitted Langmuir model with lower adsorption energetics compared with those values derived from dye adsorption over p(AA-co-MMA). The CMC/p(AA-co-MMA) exhibits a spontaneous exothermicity adsorption profile, where safranine molecules were well-oriented and adequately attached to the -OH groups of CMC by offering hydrogen bond interaction with the amine groups of dye molecules. Also, CMC/p(AA-co-MMA) possesses advanced adsorption kinetics fitting pseudo-first and second-order models. The intimate interaction between CMC and the copolymer matrix most probably impaired the pore structure of the copolymer, thus provoking the external particle diffusion rates at the expense of the internal diffusion rates. From a different angle, CMC/p(AA-co-MMA) hybrid polymer composite efficiently emerged as a promising candidate for the stabilization of metallic Ag particles, heightening their bioactive nature against *E. coli* and *S. aureus* capable of being a competitor for traditional antibiotics.

On the whole, the fabrication of hybrid polymer composites of rational hydrophilic/hydrophobic nature may hopefully become one of the most compelling issues of concern in future that possesses a generous benefaction in the field of decontamination of wastewater from organic pollutants and wastewater disinfection technologies.

## Electronic supplementary material

Below is the link to the electronic supplementary material.

Supplementary Material 1

## Data Availability

The datasets used and analyzed during the current study available from the corresponding author on reasonable request.
