# Supplementary material for: Carboxymethyl cellulose assisted reforming of poly acrylic acid co methyl methacrylate composite for wastewater treatment and effective hosting of antimicrobial silver
Source: Sci Rep. 2025 Feb 8;15:4731. doi: 10.1038/s41598-025-86214-5 (PMC11807114; doi:10.1038/s41598-025-86214-5)
Supplement: Supplementary file 1 — Supplementary Material 1 [file 41598_2025_86214_MOESM1_ESM.docx]

Supplementary file

Carboxymethyl cellulose assisted reforming of poly acrylic acid co methyl methacrylate composite for wastewater treatment and effective hosting of antimicrobial silver

Ahmed Hamdy ^*,a ,b,c^, Hassan Nageh ^b^, S. A. Hassan ^a^,  [Mohamed A. Mekewi](http://www.tandfonline.com/action/doSearch?action=runSearch&type=advanced&searchType=journal&result=true&prevSearch=%2Bauthorsfield%3A%28Mekewi%2C+M+A%29)^a^, Atef S. Darwish ^*,a^

^a^ Department of Chemistry, Faculty of Science, Ain Shams University, Cairo 11566, Egypt.

^b^ Nanotechnology Research Centre (NTRC), The British University in Egypt, Cairo 11837, Egypt.

^c^ Faculty of Dentistry, The British University in Egypt, Cairo 11837, Egypt.

* Email; [ahmed.mokhtar@bue.edu.eg](mailto:ahmed.mokhtar@bue.edu.eg), [atef_mouharam@sci.asu.edu.eg](mailto:atef_mouharam@sci.asu.edu.eg)

Supplementary data

| 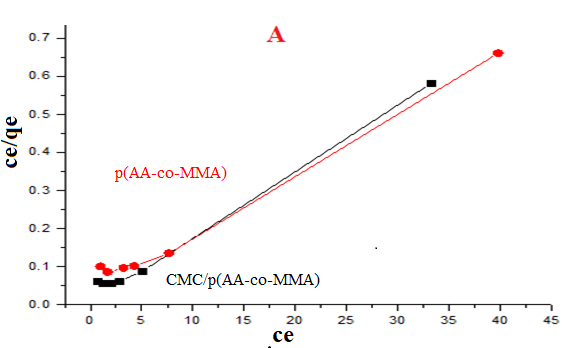 | 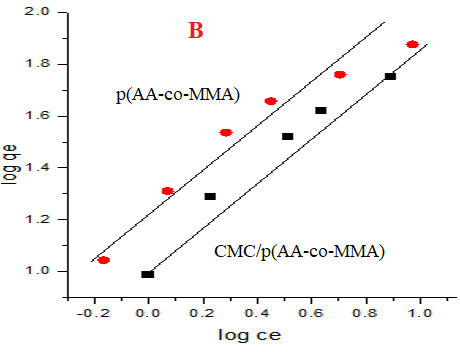 | |
| --- | --- | --- |
| 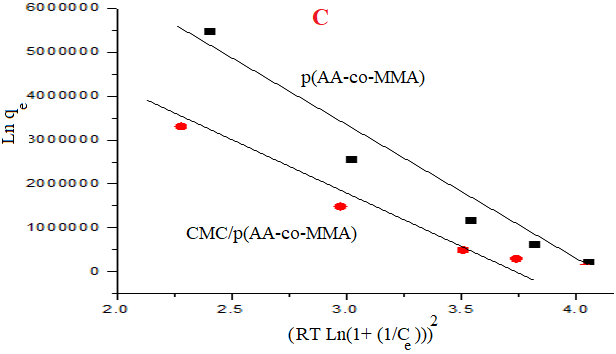 | |  |

**Fig. S1:** Langmuir (A), Freundlich (B), and Dubinin–Radushkevich (C) adsorption isotherm plots for removal of safranine dye from wastewater using p(AA-co-MMA) copolymer and CMC/p(AA-co-MMA) hybrid polymer composite (pH: 7.8; sorbent content 2 g/L; initial dye concentrations 10, 20, 40, 90, 120, 160 mg/L; temperature 25^o^C; contact equilibrium time: 3 h).

| 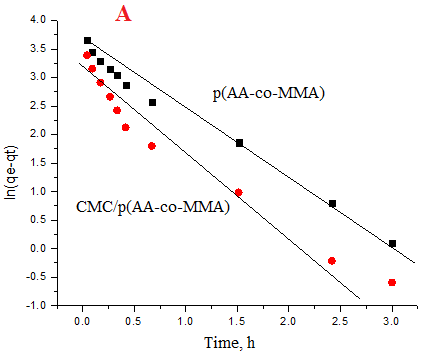 | 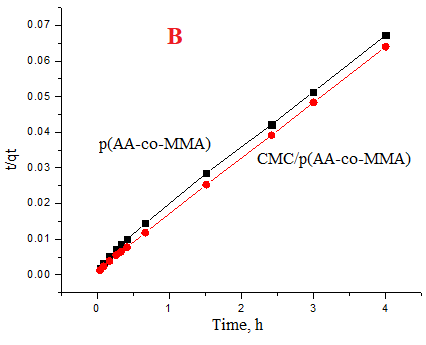 |
| --- | --- |
| 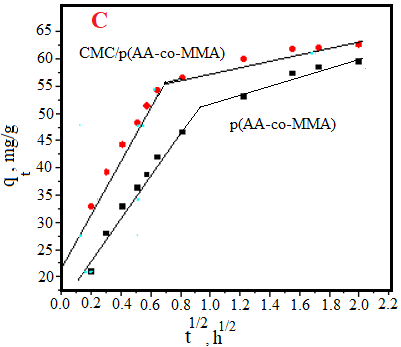 | |

**Fig. S2:** (A) pseudo-first-order (A), pseudo-second-order (B), and intraparticle diffusion (C) kinetic plots of safranine dye removal from wastewater using p(AA-co-MMA) copolymer and CMC/p(AA-co-MMA) hybrid polymer composite (pH: 7.8; sorbent dose 2 g/L; initial dye concentrations 120 mg/L; temperature 25^o^C).


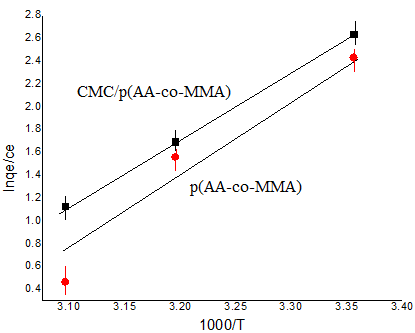


**Fig. S3:** Thermodynamic equilibrium plot for the adsorption of safranine dye over p(AA-co-MMA) copolymer and CMC/p(AA-co-MMA) hybrid polymer composite from wastewater. Conditions: pH: 7.8; sorbent dose 2 g/L; initial dye concentration: 120 mg/L; temperature 25^o^C).

**Table S1**: Abbreviations of terms used in the present study.

| Terms | Abbreviations |
| --- | --- |
| CMC | Carboxymethyl cellulose. |
| AA | Acrylic acid. |
| MMA | Methyl methacrylate. |
| KPS | Potassium persulfate. |
| NMBA | N, N-methylene-bis(acrylamide). |
| PAA | Poly acrylic acid. |
| PMMA | Polymethyl methacrylate. |
| p(AA-co-MMA) | Poly (acrylic acid-co-methyl methacrylate) copolymer. |
| CMC/p(AA-co-MMA) | Poly (acrylic acid-co-methyl methacrylate) copolymer hybridized with carboxymethyl cellulose (hybrid polymer composite). |
| Ag@p(AA-co-MMA) | Silver embedded onto poly (acrylic acid-co-methyl methacrylate) copolymer. |
| Ag@CMC/p(AA-co-MMA) | Silver embedded onto poly (acrylic acid-co-methyl methacrylate) copolymer hybridized with carboxymethyl cellulose. |
| S | Swelling percentage. |
| R | Removal percentage of dye from wastewater. |
| q_e_ | Equilibrium dye adsorption capacity of materials under study. |
| q_t_ | Dye adsorption capacity of materials under study at different time intervals. |
| q_D_  K_f_  q_m_ | Saturation adsorption capacity of materials under study.  Freundlich coefficient.  Monolayer adsorption capacity of materials under study. |
| R_L_  E | Dimensionless constant separation term.  Apparent free energy |
| k_1_ | Pseudo-first-order rate constant. |
| k_2_ | Pseudo-second-order rate constant. |
| k_i_ | Intra-particle diffusion rate constant. |
| R  T | General gas constant.  Temperature during the adsorption process. |
| ΔH° | Molar enthalpy change. |
| ΔS° | Molar entropy change. |
| ΔG^o^ | Gibbs free energy change. |
| ± SD | Standard deviation. |
| *E. Coli* | *Escherichia coli* NCTC10418. |
| *S. aureus* | *Staphylococcus aureus* ATCC6538. |
| R_B_ | Percentage of bacteria reduction. |
